# Induction of ischemic stroke in awake freely moving mice reveals that isoflurane anesthesia can mask the benefits of a neuroprotection therapy

**DOI:** 10.3389/fnene.2014.00001

**Published:** 2014-04-03

**Authors:** Angela Seto, Stephanie Taylor, Dustin Trudeau, Ian Swan, Jay Leung, Patrick Reeson, Kerry R. Delaney, Craig E. Brown

**Affiliations:** ^1^Division of Medical Sciences, University of VictoriaVictoria, BC, Canada; ^2^Department of Biology, University of VictoriaVictoria, BC, Canada; ^3^Department of Psychiatry, University of British ColumbiaVancouver, BC, Canada

**Keywords:** anesthesia, freely moving, neuroprotection, nicotinic receptor, stroke

## Abstract

Anesthetics such as isoflurane are commonly used to sedate experimental animals during the induction of stroke. Since these agents are known to modulate synaptic excitability, inflammation and blood flow, they could hinder the development and discovery of new neuroprotection therapies. To address this issue, we developed a protocol for inducing photothrombotic occlusion of cerebral vessels in fully conscious mice and tested two potential neuroprotectant drugs (a GluN2B or α4β2 nicotinic receptor antagonist). Our data show in vehicle treated mice that just 20 min of exposure to isoflurane during stroke induction can significantly reduce ischemic cortical damage relative to mice that were awake during stroke. When comparing potential stroke therapies, none provided any level of neuroprotection if the stroke was induced with anesthesia. However, if mice were fully conscious during stroke, the α4β2 nicotinic receptor antagonist reduced ischemic damage by 23% relative to vehicle treated controls, whereas the GluN2B antagonist had no significant effect. These results suggest that isoflurane anesthesia can occlude the benefits of certain stroke treatments and warrant caution when using anesthetics for pre-clinical testing of neuroprotective agents.

## Introduction

Anesthetics are commonly used in humans undergoing neurosurgery and experimental animals subjected to various kinds of ischemic stroke. Over the years there has been a growing appreciation that anesthetics have widespread effects on neuronal and vascular function. For example, inhaled anesthetics such as isoflurane can augment GABA, glutamate and nicotinic neurotransmission, mitochondrial function, apoptosis, inflammation and cortical blood flow (Eger, 1981; McPherson et al., 1994; Flood et al., 1997; De Sousa et al., 2000; Yamakura and Harris, 2000; Wu et al., 2012; Zhang et al., 2012; Hofacer et al., 2013; Kotani and Akaike, 2013; Altay et al., 2014). This is a potential concern for pre-clinical studies of cerebral neuroprotection since many potential treatments target the same signaling pathways as those affected by anesthesia. The effects of anesthesia on brain function can last for hours, even after the animal regains consciousness (Kapinya et al., 2002b; Saab et al., 2010), which raises the possibility that anesthesia could affect the outcome of a neuroprotection study.

In order to address this question, one needs to compare potential therapies in a model of stroke that can be induced without anesthesia. There are a few published studies that describe procedures for inducing stroke in awake animals using intracortical endothelin injection or intra-arterial infusion of microspheres (Demura et al., 1993; Gelb et al., 2002). While useful, these methods require invasive procedures such as the implantation of a brain cannula which can augment cerebral blood flow and induce inflammation. In the present study we developed a protocol for inducing focal ischemic stroke in fully awake mice using the photothrombotic method (Watson et al., 1985). An advantage of this approach is that ischemic stroke can be targeted to a specific brain region or artery (Nishimura et al., 2006; Schaffer et al., 2006; Sigler et al., 2008) without extensive surgical training or invasive procedures. Here we show that not only can isoflurane anesthesia reduce the extent of ischemic damage after stroke, but it can mask the protective effects of a stroke therapy.

## Materials and methods

### Animals

One hundred and fifty-six adult (2–4 months old) male wild-type and YFP-H line (Feng et al., 2000) mice with C57BL/6J background were analyzed in this study. All experiments were approved by the University of Victoria's Animal Care Committee and adhered to guidelines set by the Canadian Council for Animal Care.

### Apparatus and procedure for inducing stroke

**Figure 1:**
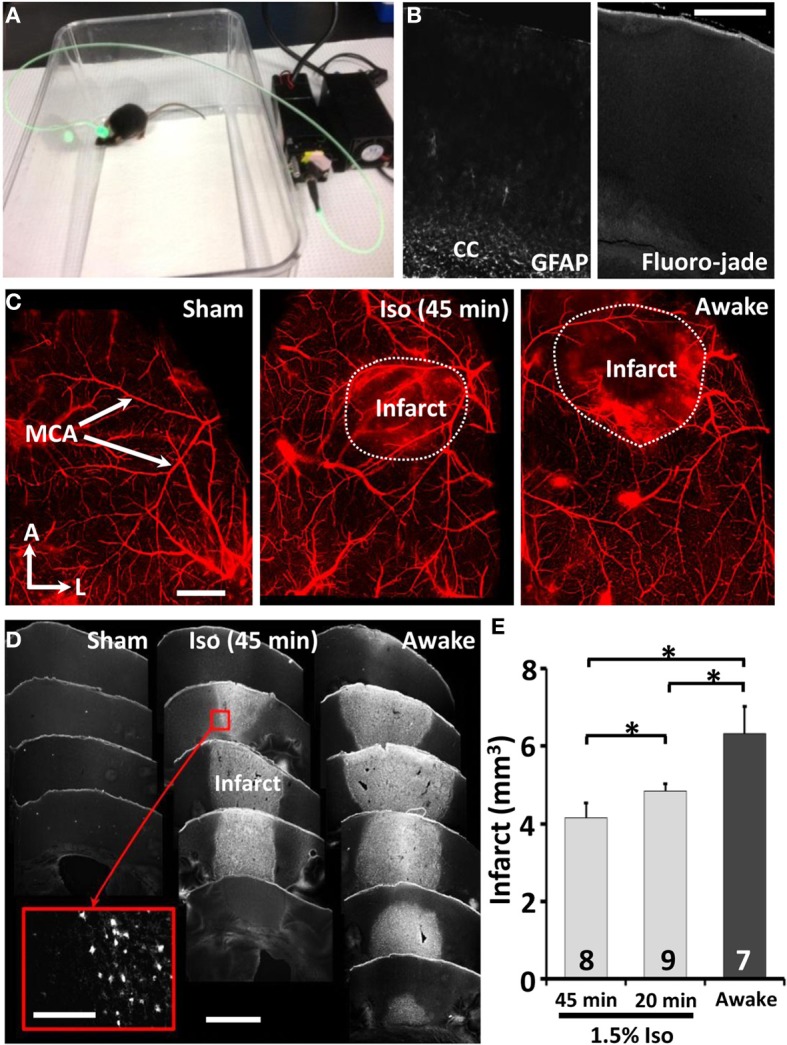
**Twenty minutes of isoflurane reduces the extent of ischemic damage. (A)** Picture of an awake freely moving mouse during induction of photothrombotic stroke. **(B)** Confocal images of brain sections 24 h after sham stroke procedure indicates little to no reactive astrocytes (GFAP staining, left panel) or cell death (FJC, right panel) near the cortical surface where the skull was thinned. We should note that our positive controls (perforating the skull and surface vasculature with the dental drill) indicated that GFAP and Fluoro-jade staining were sensitive to revealing tell tale signs of cortical damage. Scale bar = 0.5 mm. **(C)** Whole mount confocal images of surface vasculature labeled with Evans blue dye 24 h after sham procedure or induction of stroke in mice anesthetized with 1.5% isoflurane for 45 min or without (awake group). Scale bar = 1 mm **(D)** Images showing coronal brain sections stained with Fluorojade C for ischemic cell death. Scale bar = 1 mm. Scale bar for inset = 100 μm (**E**) Quantitative analysis of infarct volume 24 h after stroke. ^*^p ≤ 0.05.

**Figure 2:**
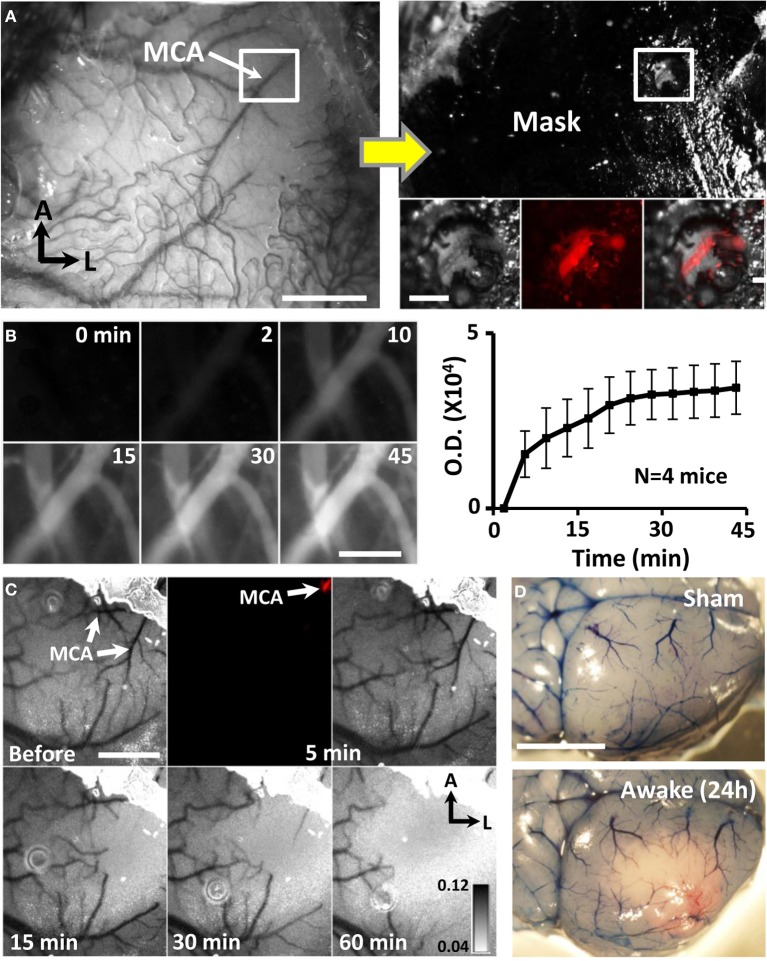
**Targeted ischemic stroke of the distal MCA in awake mice. (A)** Photomicrograph of distal MCA through the intact mouse skull. Adjacent image shows skull after masking procedure to restrict photoactivation to the distal MCA. Inset boxes show the segment of the MCA (labeled with Texas red dextran) that will be subjected to photoactivation. Scale bar = 1 mm. Scale bar in boxed region = 0.2 mm. **(B)** Images showing Rose Bengal dye fluorescence in surface vessels following dye injection. Images and optical density plot of dye fluorescence in vessels show that the dye circulated in the blood for at least 45 min. Scale bar = 0.2 mm. **(C)** Transcranial laser speckle contrast images of cortical blood flow at baseline and during 5, 15, 30, and 60 min of photoactivation. Darker tones correspond to regions and vessels with higher blood flow. Fluorescence image shown at 5 min illustrates selective photoactivation of Rose Bengal dye in a distal branch of the MCA (2nd panel). Scale bar = 1 mm. **(D)** Images show surface vasculature labeled with Evans blue dye 24 h after sham operation or stroke induction while awake. Scale bar = 3 mm.

We used either a 20 or 200 mW 532 nm diode laser (Beta Electronics) for photothrombotic occlusion of surface vessels (Figure 1) or targeted occlusion of the distal middle cerebral artery (MCA, Figure 2). The laser was focused onto the polished end of an unjacketed 1 mm plastic optical fiber (Edmund Scientific, 02534) mounted in an FC-style optical connector (Thorlabs, 3012662-1050) using an 18 mm focal length FC compatible collimator assembly (Thorlabs, F280FC-A). Output power at the fiber tip was 12 mW (15.29 mW/mm^2^) for the 20 mW laser. For the 200 mW laser, a neutral density filter was used to attenuate the laser output power from 104 to 25 mW (31.85 mW/mm^2^) at the fiber tip. The polished output end of the fiber was mounted in a customized male Luer connector, which mated with a corresponding female Luer connector cemented to the skull.

In order to prepare mice for the induction of stroke on the following day (to install the headcap for the fiber optic attachment), all animals were anaesthetized with 1.5% isoflurane mixed with medical air (20% Oxygen, 80% Nitrogen) at a flow rate of 0.7L/min. Since prolonged exposure to isoflurane anesthesia (3 h) can be neuroprotective (Kapinya et al., 2002b), we restricted surgery to 15–20 min duration. Body temperature was maintained at 37°C during surgery using a rectal thermoprobe and a temperature feedback regulator. For non-specific photothrombotic occlusion of surface vessels (see Figure 1), a 2 mm diameter circular region of skull overlying the right forelimb somatosensory cortex was thinned using a high speed dental drill. For targeted distal MCA occlusion (Figure 2), a branch of the distal MCA in the right hemisphere (4 mm lateral to bregma) was first visualized by moistening the skull with sterile buffer. The skull directly over a single branch of the MCA was thinned using a 0.4 mm diameter carbide burr (FG ¼ Henry Schein). Areas of skull adjacent to the thinned region of skull were masked with permanent black marker. Control experiments indicated that a photothrombotic stroke could not be induced through the masked regions. A female luer lock connector (diameter 6 mm) was fixed to the skull with cyanoacrylate glue and dental cement. All surgeries were performed blind to condition. Mice were briefly warmed under a heat lamp and returned to their home cages.

**Figure 3:**
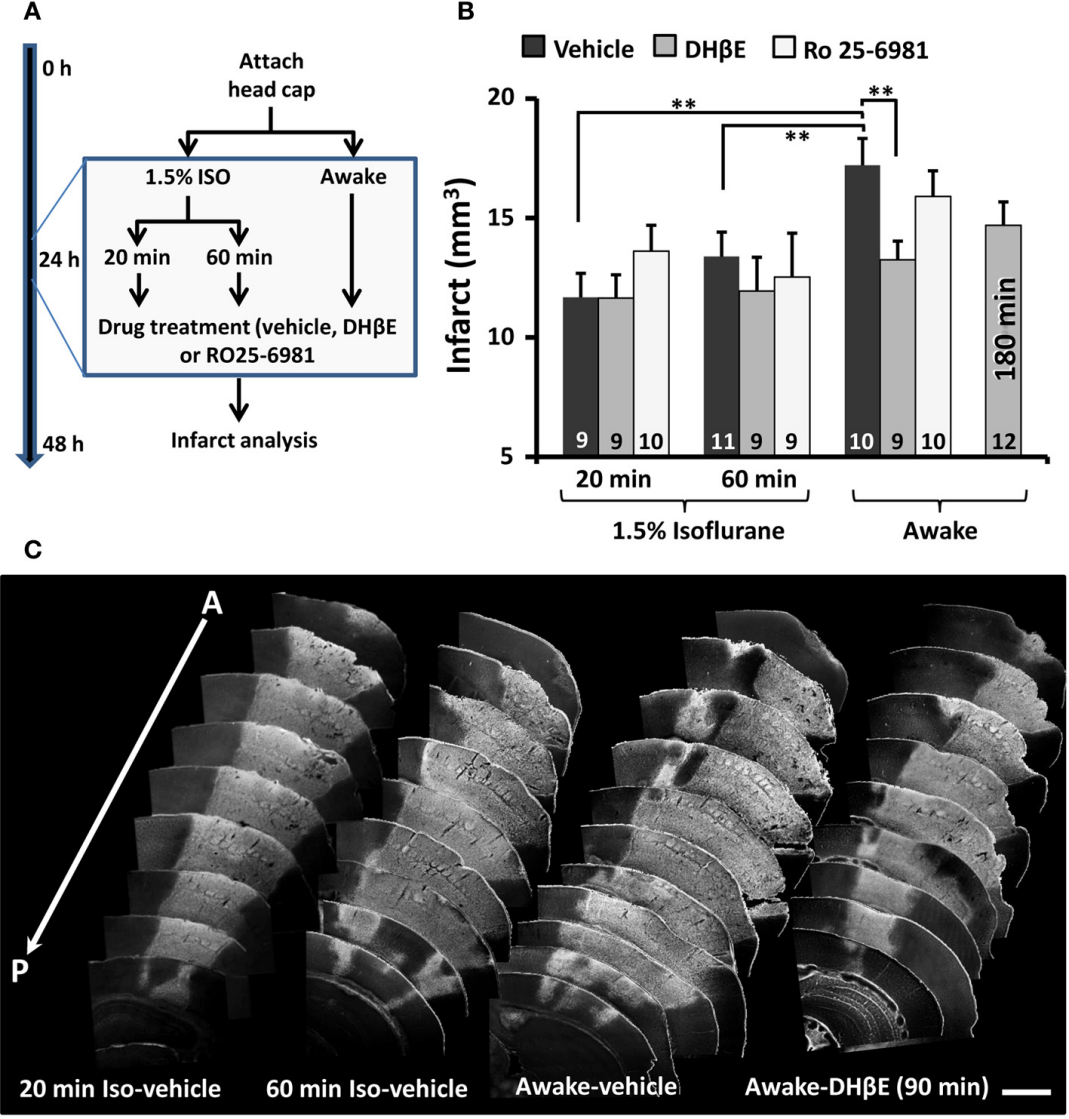
**The neuroprotective effects of an α4β2 nAChR antagonist (DH βE) are apparent only when stroke is induced without anesthesia. (A)** Diagram summarizing neuroprotection experiments. **(B)** Histogram shows the effects of vehicle, DHβE and Ro25-6981 administered 1.5 h after the initiation of photothrombosis on infarct volumes assessed 24 h after stroke. Note that DHβE treatment was also examined 180 min after stroke. The number of mice in each group is indicated in each bar. ^**^p ≤ 0.01. **(C)** Anterior to posterior arrangement of representative coronal brain sections stained with Fluorojade **(C)**. Fluorescent (white) staining demarcates the cerebral infarction. Scale bar = 1 mm.

**Figure 4:**
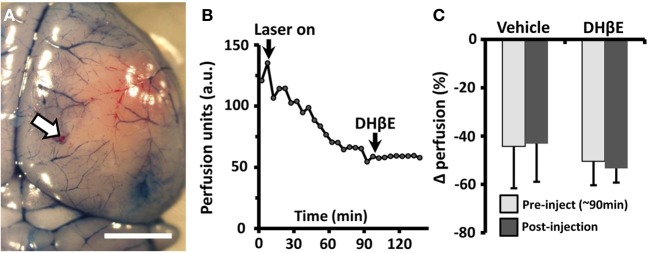
**The neuroprotective effects of DHβE in awake mice are not explained by changes in penumbral blood flow. (A)** Photomicrograph showing the site of transcranial laser doppler recording on the cortical surface (site marked with DiI, white arrow) 180 min after photo-occlusion of the MCA. Evans blue dye was injected to visualize the ischemic territory. Scale bar = 2 mm. **(B)** Representative transcranial laser doppler recording of blood flow in the cortical penumbra (~3.5 mm from site of distal MCA occlusion) in an awake mouse treated with DHβE at 90 min. Each data point represents a 5 min average of perfusion. Note the progressive drop in blood flow with the start of laser induced photothrombosis (“laser on”) and that DHβE has no effect on cortical perfusion. **(C)** Histogram showing the average percent change in penumbral blood flow relative to baseline perfusion in awake animals treated with vehicle (*n* = 4 mice) or DHβE (*n* = 5 mice). On average, neither vehicle or DHβE injection altered blood perfusion in the penumbra relative to pre-injection values.

Twenty four hours after the headcap was installed, animals were randomly assigned to one of the experimental groups. To enhance transparency of the skull and photoactivation, 20 μl of mineral oil was pipetted into the luer lock before connecting the fiber optic cable. This was followed by an injection of 1% Rose Bengal dye (110 mg/kg, i.p.) dissolved in HEPES buffered ACSF. Photoactivation was initiated using a 532 nm laser for 15 min in our first set of experiments (Figure 1) or 60 min for targeted photothrombotic occlusion of the MCA (Figures 2–4). Mice that did not show a permanent obstruction of the MCA (as shown with Evans Blue dye 24 h after stroke) were excluded from the study. Furthermore, 2 of 41 mice appeared to have a seizure 1–2 h after stroke (1 treated with DHβE, the other vehicle) and were immediately removed from the study.

### Drugs

Ninety minutes after the start of photothrombosis, mice received a single injection (i.p.) of the GluN2B receptor antagonist Ro25-6981 (6 mg/kg, Tocris Bioscience), the α4β2 specific nAChR antagonist dihydro-β-erythroidine hydrobromide (3 mg/kg DHβE, Tocris Bioscience) or 0.9% saline (vehicle). An additional group of mice was injected with 3 mg/kg DHβE 180 min after stroke. Previous studies have shown that both drugs readily cross the blood brain barrier and alter cortical excitability (Liu et al., 2007; Brown et al., 2012).

### Evans blue angiograms and analysis

Mice were administered 0.2 mL of 4% Evans blue dye (i.v., dissolved in saline), briefly anesthetized with 2% isoflurane and decapitated 5 min later. Paraformaldehyde fixed brains were imaged at 4× magnification (*NA* = 0.13) with an Olympus confocal microscope. We used 515 and 635 nm lasers to excite rose bengal and Evans blue, respectively. Image stacks were collected using a Kalman filter (average of 2 images) in 25 μm *z*-steps at 640 × 640 pixels (5 μm/pixel) with a pixel dwell time of 4 μs.

### Infarct volume analysis

Brains were sectioned at 50 μm on a Leica vibratome in the coronal plane. To quantify infarct volume 24 or 72 h after stroke, every third section (150 μm apart) was stained with 0.0001% Fluoro Jade C (FJC) (Gu et al., 2012). Wide-field images of FJC stained sections were collected with a 4× objective (*NA* = 0.13) using a GFP filter set on an upright Olympus microscope. Using NIH Image J software (version 1.44d) the infarct area showing dense fluorojade staining was outlined in each image by an observer blind to condition. Infarct volume was calculated by summing up the infarct area for each section multiplied by the distance between each section (150 μm).

### Laser speckle contrast imaging

A 785 nm laser with 2 mW output power (ThorLabs) was directed at the cortex at a 30° angle (Zhang and Murphy, 2007; Armitage et al., 2010). Blood flow was assessed by collecting 50 images (696 × 520 pixels, 6.4 μm/pixel) with 5–10 ms exposure over a 5 s period. Images were processed with a variance filter (radius = 1) and then averaged together. A single image showing the standard deviation was generated by taking the square root of the averaged (variance processed) image. To correct for uneven illumination, the standard deviation image was then divided by the mean of all the raw images.

### Transcranial laser doppler recordings in awake mice

A 0.5 mm plastic fiber optic coupled to a laser doppler blood flow monitor (Moor Instruments, moorVMS-LDF1) was threaded inside a 20 gauge cannula (attached to the Luer lock on the skull) and fixed in place with cyanoacrylate glue. Perfusion measurements were collected at 10 Hz over 150 min period. Perfusion measurements were typically 3–4 mm posterior and medial from the segment of MCA that was photo-activated. This cortical region was at the interface between ischemic and non-ischemic territory and hence denoted as “penumbra.” After 10 min of baseline measurements, stroke was initiated as described above. Ninety minutes later, mice were injected with DHβE (3 mg/kg) or vehicle. Laser Doppler data were importing into IGOR Pro (Wavemetrics, Eugene OR) and processed with a differentiation algorithm followed by manual thresholding and binomial smoothing (radius = 3) to help eliminate movement related data artifacts. Perfusion values for each experiment were generated by taking the average perfusion over a 10 min period before the induction of stroke, before injection (see Figure 4C, “pre-inject”) or 5–15 after injection (“post-inject”).

### Statistics

The effects of anesthesia and drug treatment were assessed using an Analysis of Variance (ANOVA). Statistical comparisons between groups were conducted using independent samples *t*-tests. A Bonferroni correction was used for multiple unplanned *post-hoc* comparisons. Based on preliminary variances using this new model of stroke (assuming a significance level of 0.05 for a 2-sided test, 80% power), we calculated that a sample size of at least 6 mice was necessary for experiment 1 where regional photoactivation of somatosensory cortex was employed, whereas 9 mice were needed for experiments using targeted MCA occlusion. A Chi-squared analysis was used to test for significant differences in stroke success and mortality rates. Data are presented as the mean ± s.e.m.

## Results

In our first set of experiments, we used the photothrombotic method to regionally occlude cortical surface vessels over the primary forelimb somatosensory cortex (Figure 1A). Mice were randomly assigned to receive either sham stroke (laser exposure without dye injection or vice versa) or stroke while awake (*n* = 7) or anesthetized with 1.5% isoflurane for 20 (*n* = 9) or 45 min. Consistent with previous studies (Brown et al., 2009; Grutzendler et al., 2011), thinning the skull or exposure to the laser in sham operates did not induce reactive gliosis or cell death near the cortical surface (*n* = 3, Figure 1B). As shown in surface angiograms (Figure 1C) and Fluoro-Jade C stained brain sections (Figure 1D), mice that were awake during stroke had a significantly larger infarction (Figure 1E) than those anesthetized for 20 [*t*_(7)_ = 2.04, *p* < 0.05] or 45 min [*t*_(9)_ = 2.72, *p* < 0.05]. Control experiments imaging the spread of light from the fiber optic tip ruled out the possibility that larger infarcts in awake ambulatory mice was caused by subtle movement of the fiber optic attachment and hence a greater area of photoactivation. Furthermore by measuring Rose Bengal fluorescence in the blood plasma 20 min after injection, we determined that anesthesia did not significantly affect its absorption into the bloodstream [*t*_(15)_ = 0.31, *p* = 0.38]. These results demonstrate that even brief exposure to isoflurane during stroke induction reduces the extent of ischemic damage.

In humans, focal ischemic stroke typically involves a thrombo-embolic occlusion of a specific segment of the MCA rather than simultaneous generation of thrombotic clots in many penetrating arterioles and micro-vessels over a cortical region (Carmichael, 2005; Jackman et al., 2011). Therefore to create a more realistic model of ischemic stroke that could be used for neuroprotection studies, we restricted photoactivation to a single segment of the distal MCA by first thinning a 0.4 mm diameter region of skull directly over the MCA and then masking adjacent areas of the skull (Figure 2A). Consistent with previous studies that have varied occlusion duration (Morancho et al., 2012), our pilot experiments indicated that 60 min of photoactivation, but not 15–30 min (*n* = 6 mice) was required to reliably induce a permanent MCA occlusion. Longer periods of photoactivation were made possible by the fact that dye circulated in the blood for at least 45 min after injection (Figure 2B). Laser speckle imaging confirmed that blood flow was progressively reduced in branches downstream of the MCA after the start of photoactivation (Figure 2C). In order to illustrate the extent of ischemic cortical tissue in the right hemisphere, Evans blue dye was injected 24 h after stroke (Figure 2D). Dye injection was useful for verifying the presence of a permanent occlusion which was evident by the absence of circulating dye near the site of MCA photoactivation. Our success rate for inducing targeted MCA stroke in awake vs. anesthetized animals was 91.5 and 80.0% respectively (χ2 = 1.18, *p* > 0.05). The mortality rate was 5.6% for awake mice and 2.2% for anesthetized mice (χ2 = 4.68, *p* < 0.05). It should be noted that mice subjected to stroke while awake did not show any overt signs of pain or distress such as vocalizations, head scratching or aggressiveness. Interestingly, all mice with a confirmed permanent MCA occlusion (verified post-mortem) showed a reduction in ambulatory behavior at the time of photoactivation. This behavioral pattern was useful to provide real time information about the success of the stroke procedure.

Since distal MCA occlusion is often used in neuroprotection studies, we asked whether the efficacy of a stroke therapy depends on whether the stroke was induced in an awake or anesthetized animal. Accordingly, mice that were awake or anesthetized with isoflurane (1.5% in air) for 20 or 60 min were given either a single intraperitoneal injection of a GluN2B receptor antagonist Ro 25-6981 (6 mg/kg), an α4β2 nicotinic acetylcholine receptor (nAChR) antagonist DHβE (3 mg/kg) or vehicle 90 min after the start of the MCA occlusion (Figure 3A). We reasoned that since ischemia stimulates glutamate and acetylcholine release (Dirnagl et al., 1999; Kiewert et al., 2010), and that isoflurane can alter glutamate receptor signaling and block α4β2 nAChRs (Flood et al., 1997; Yamakura and Harris, 2000), it is possible that the neuroprotective effects of these drugs could have been missed in previous studies. Consistent with our first set of experiments, mice that were awake during stroke induction and treated with vehicle had significantly larger infarcts than vehicle treated mice anesthetized for 20 [*t*_(17)_ = 3.65, *p* = 0.001] or 60 min [*t*_(19)_ = 2.52, *p* = 0.01; Figures 3B,C see Table 1 for infarct details]. The extent of ischemic damage did not expand beyond the first 24 h, given that there was no difference between infarct volumes measured at 24 vs. 72 h in anesthetized mice [1 day = 13.4 ± 1.1 mm^3^ vs. 3 day infarct volume = 12.8 ± 1.2 mm^3^; *t*_(19)_ = 0.31, *p* = 0.38]. With respect to stroke treatments, none had any impact on infarct volume in mice that were anesthetized for either 20 [*F*_(2, 25)_ = 1.01, *p* = 0.38] or 60 min [*F*_(2, 26)_ = 0.31, *p* = 0.73] during the induction of stroke (Figure 3B). The absence of a neuroprotective effect with Ro 25-6981 treatment (in anesthetized mice) is consistent with previous studies (Liu et al., 2007; Sun et al., 2008). However there was a significant effect of drug treatment if the stroke was induced while awake [*F*_(2, 26)_ = 3.82, *p* < 0.05]. Specifically, DHβE (but not Ro 25-6981) treatment 90 min after stroke was highly effective in reducing the extent of ischemic damage relative to awake mice treated with saline [*t*_(17)_ = 2.83, *p* < 0.01; Figures 3B,C]. There was a trend toward smaller infarcts in an additional group of awake mice treated with DHβE 180 min after occlusion [*t*_(20)_ = 1.66, *p* = 0.05; Figure 3B]. These data show that just 20 min of exposure to isoflurane during stroke induction can mask the benefits of a neuroprotection therapy.

**Table 1:** **Summary of ischemic damage from each experimental group measured 24 h after targeted occlusion of the MCA**.

**Condition**	**Drug treatment**	**No. of mice**	**Total (mm^3^)**	**Cortex (mm^3^)**	**Striatum (mm^3^)**
20 min ISO	Vehicle	9	11.7 ± 1.00	11.6 ± 0.98	0.1 ± 0.06
20 min ISO	DHβE	9	11.7 ± 0.97	11.6 ± 0.95	0.1 ± 0.05
20 min ISO	Ro25-6981	10	13.6 ± 1.08	13.4 ± 1.05	0.2 ± 0.12
60 min ISO	Vehicle	11	13.4 ± 1.02	12.9 ± 1.02	0.5 ± 0.25
60 min ISO	DHβE	9	11.9 ± 1.41	11.6 ± 1.31	0.3 ± 0.18
60 min ISO	Ro25-6981	9	12.5 ± 1.62	11.8 ± 1.35	0.7 ± 0.36
Awake	Vehicle	10	17.2 ± 1.12	16.4 ± 0.79	0.8 ± 0.51
Awake	DHβE	9	13.3 ± 0.78	13.0 ± 0.75	0.3 ± 0.09
Awake	Ro25-6981	10	15.9 ± 1.06	15.1 ± 0.89	0.8 ± 0.23
Awake	DHβE (3 h)	12	14.7 ± 0.97	14.2 ± 1.00	0.5 ± 0.21

Unless indicated, all drug treatments were administered 90 min after the initiation of photothrombosis. All values are expressed as the mean ± s.e.m.

In order to determine whether the neuroprotective effects of DHβE in awake mice could be explained by increased blood flow in the cortical penumbra, we transcranially recorded blood flow in awake mice using laser doppler flowmetry (Figures 4A,B). Our analysis indicated that blood flow in the cortical penumbra was reduced by 44 ± 17% in vehicle and 50 ± 10% in DHβE treated mice 90 min after initiating photothrombosis (Figures 4B,C). Treatment with DHβE did not significantly alter blood flow in the penumbra relative to pre-injection levels [Figure 4C, *t*_(8)_ = 0.10, *p* = 0.46]. Furthermore, there were no significant differences between saline and DHβE treated mice in heart rate, respiration, blood gas/electrolyte concentrations or blood pH (Table 2; *n* = 3 per group, *p* > 0.05 for all comparisons). Therefore the neuroprotective effects of DHβE in awake mice are not explained by differences in penumbral blood flow or global changes in several cardiovascular parameters.

**Table 2:** **Physiological variables for drug experiments**.

	**Awake—vehicle**	**Awake—DH βE**
pH	7.35 ± 0.04	7.32 ± 0.04
pCO_2_ (mmHg)	38.3 ± 0.5	37.6 ± 0.9
pO_2_ (mmHg)	89.6 ± 3.8	95.3 ± 7.8
HCO_3_ (mmol/L)	21.2 ± 1.9	19.3 ± 1.3
Na (mmol/L)	146 ± 1.2	148 ± 0.3
K (mmol/L)	6.2 ± 0.3	5.6 ± 0.2
Glucose (mmol/L)	240 ± 26	204 ± 38
Hematocrit (%)	46.3 ± 2.3	45.2 ± 2.0
Hemoglobin (g/dL)	15.8 ± 2.3	15.4 ± 0.7
O_2_ saturation (%)	94.2 ± 1.4	92.0 ± 1.7
Heart rate (bpm)	671 ± 60	702 ± 13
Breath rate (brpm)	151 ± 16	183 ± 16

Summary of physiological variables measured for drug therapy experiments. Arterial blood was measured with an iSTAT CG8+ analyzer in un-anesthetized mice 30 min after administration of vehicle (n = 3) or DHβE (3 mg/kg, n = 3). Independent samples t-tests were used to compare groups. None of the variables were significantly different based on an alpha level of 0.01. Data are presented as the mean ± s.e.m.

## Discussion

The goal of the present study was to address two key questions: (1) does isoflurane anesthesia affect the extent of ischemic stroke damage and (2) does the presence of isoflurane anesthesia during stroke induction influence how well a neuroprotective therapy appears to work? In order to investigate the first question, we needed to develop a method for inducing stroke that eliminated the need for anesthetics. Photothrombosis was a logical choice for this given that the photosensitive dye could be administered through an intraperioneal injection, while excitation light could be delivered in a relatively non-invasive manner through a fiber optic assembly. Our stroke protocol provides certain advantages over other commonly used experimental models of focal ischemia (Longa et al., 1989). Most obvious is the elimination of anesthesia which necessarily complicates the study of how stroke or a neuroprotective treatment affects brain function, circulation, and viability. Using un-anaesthetized animals also opens up the possibility for examining how certain behavioral states (sleep-wakefulness, satiety, exercise) can affect ischemic damage. Another advantage is that no craniectomy is required (Sugimori et al., 2004). This reduces the likelihood of craniectomy induced hemorrhage and infection which can occur using endothelin, thermal or mechanical occlusion of the MCA. More practical advantages include the speed and ease with which the stroke can be performed. In our lab, 3 different experimenters were able to become proficient in this method of stroke in just a few practice attempts. It should also be noted that the success rate for inducing stroke was quite high, particularly in awake mice (~92%). Moreover, the co-efficient of variation for infarct volumes in awake mice was 20.5%, suggesting that ischemic damage was highly reproducible. Given some of the advantages outlined above and the fact that targeted photothrombotic occlusion of the MCA induces a penumbral region of reduced cortical blood flow (see Figures 4B,C) (Yao et al., 2003), the present model could be very useful for future neuroprotection studies.

As is the case with any model of stroke, there are limitations and potential caveats with our approach. For one, our stroke protocol still requires 15–20 min of exposure to anesthesia to thin the skull and attach the headcap. Since 3 h of isoflurane exposure can affect stroke damage when induced 24 h later (Kapinya et al., 2002a), there is a possibility that this exposure could affect our results. However, this scenario seems unlikely since all stroke groups (awake and anesthetized) were briefly exposed to isoflurane and we were still able to detect a robust difference in infarct volumes between awake and anesthetized mice. Second the mice need to be tethered to the light weight fiber optic for 60 min to ensure a permanent occlusion of the MCA. Therefore it is possible that the fiber optic attachment might induce a stress response or suppress some natural behaviors. While a potential concern, we noticed in mice with a sham stroke or those few instances where we were unsuccessful inducing the stroke, mice showed normal exploratory and grooming behaviors during the 60 min photoactivation period. It should also be noted that no signs of cell death were observed in these mice, therefore any heat associated with the laser was not sufficient to induce damage which is consistent with previous studies using photothrombosis (Sugimori et al., 2004; Sweetnam et al., 2012). Future iterations of the present model could incorporate a 568 nm laser beam (Sugimori et al., 2004) which more efficiently excites Rose Bengal dye thereby reducing the time needed for photoactivation. Another potential drawback is that photo-activation of Rose Bengal dye generates singlet oxygen which directly damages the vascular endothelium. This can lead to a rapidly progressing infarct and vasogenic edema (Watson et al., 1985; Witte, 2010). In humans these events are usually delayed a few hours, therefore how closely photochemically induced ischemic damage models the human condition is unclear (Witte, 2010). Furthermore, since photothrombosis leads to a permanent irreversible occlusion, the ability for many neuroprotectant drugs to promote re-perfusion into the ischemic core may be limited.

Isoflurane was chosen in the present study because of its widespread use in animal studies of stroke. However, isoflurane and other inhaled anesthetics are well known to have multiple effects on brain function. For example, isoflurane dose dependently enhances GABAergic inhibitory post-synaptic currents, while inhibiting glutamate release and AMPA mediated receptor currents (Flood et al., 1997; Larsen and Langmoen, 1998; De Sousa et al., 2000; Saab et al., 2010; Kotani and Akaike, 2013). Isoflurane has also been shown to dilate cerebral vessels and increase cortical blood flow (Eger, 1981; McPherson et al., 1994; Iida et al., 1998). Given these diverse effects, it is plausible that isoflurane could have intrinsic neuroprotective properties. However, there is some debate in the literature on this issue. For example, Nehls et al. (1987) reported that monkeys anesthetized with isoflurane were more likely to suffer a stroke and resultant hemiplegia than those treated with barbiturates. Contrasting with these results, a retrospective study of patients undergoing cardiac surgery revealed that those anesthetized with isoflurane had a reduced risk of ischemic damage compared to those exposed to enflurane or halothane (Michenfelder et al., 1987). Experimental models of cerebral ischemia have found more consistent results in that inhaled anesthetics such as isoflurane and sevoflurane attenuate stroke damage and reduce the severity of neurological deficits (Warner et al., 1993; Sakai et al., 2007; Traystman, 2010; Zhou et al., 2010). One potential limitation of past studies was that the effects of isoflurane were not compared to an animal that was completely free of anesthetic on the day stroke was induced. Using both regional and targeted occlusion of cerebral vessels in awake and anesthetized mice, our data support the hypothesis that isoflurane can protect the brain from ischemic damage. While we cannot extrapolate our findings to other anesthetics, future studies utilizing the awake stroke model could help with this determination.

With respect to our second objective, our data clearly indicate that the efficacy of a drug therapy is in some cases dependent on whether the stroke was induced with or without a general anesthetic. To our knowledge, this is the first study to show such an effect. Moreover, no previous study has shown that blocking α4β2 nicotinic receptors *in vivo* can protect the brain from ischemic damage. Given that isoflurane is a potent inhibitor of α4β2 nAChRs (Yamakura and Harris, 2000), and that most preclinical studies induce stroke with an anesthetic (often using isoflurane), it is possible that the neuroprotective effects of an α4β2 nAChR antagonist could have been missed in past studies. Indeed, we too did not find any beneficial effects of DHβE if the treatment was initiated in mice exposed to isoflurane anesthesia during stroke induction. The mechanism through which the α4β2 nAChR antagonist confers neuroprotection in awake mice remains speculative but it was not a consequence of increased collateral blood flow or changes in physiological variables such as heart rate, respiration, blood gas or electrolyte concentrations. Furthermore, a recent study demonstrated that systemic injection of DHβE (at 3 mg/kg) in adult C57BL6 mice has minimal effect on core body temperature, thus ruling out hypothermia as a cause (Rezvani et al., 2012). While beyond the scope of the present study, future experiments directed at understanding how α4β2 nAChRs modulate glutamate release/excitotoxicity (Lambe et al., 2003), spreading depression (Sheardown, 1997; Douglas et al., 2011), blood brain barrier permeability (Hawkins et al., 2005) or inflammatory responses to ischemia should help resolve this issue.

### Conflict of interest statement

The authors declare that the research was conducted in the absence of any commercial or financial relationships that could be construed as a potential conflict of interest.
